# Exploring the relationship between intestinal immunity and obesity: A bibliometric and knowledge-map analysis

**DOI:** 10.1097/MD.0000000000043790

**Published:** 2025-08-08

**Authors:** Shanshan Cui, Shuai Shang, Zihui Yan

**Affiliations:** a Department of Scientific Research and Education, Shengli Oilfield Central Hospital, Dongying, China.

**Keywords:** bibliometric analysis, intestinal immunity, obesity

## Abstract

**Background:**

This study aimed to identify influential researchers, institutions, and countries and reveal the evolution of research hotspots and themes in the field of the relationship between intestinal immunity and obesity through bibliometric analysis.

**Methods:**

We searched and selected the Web of Science database for publications on intestinal immunity and obesity between 2004 and 2024, followed by bibliometric and visualization analysis using CiteSpace, GraphPad Prism 8, Gephi, and Charticulator.

**Results:**

A total of 3333 publications involving 16,144 authors, 3372 research institutions, and 97 countries or regions were analyzed. The United States led in both total publication counts and betweenness centrality. The influential institutions in this field were the Institut national de la santé et de la recherche médicale and the University of Reading, which ranked first in publication output and betweenness centrality, respectively. Patrice D. Cani was the most influential researcher. Research on the relationship between intestinal immunity and obesity mainly focused on the pathogenesis of obesity and obesity-related diseases. Academic attention to obesity pathogenesis shifted from innate to adaptive immunity and transitioned from gut dysbiosis to microbial metabolites. Meanwhile, obesity-related diseases evolved from intestinal disorders to metabolic dysfunction-related cardiovascular diseases and liver diseases. The research themes in this field evolved through 3 stages: the early stage focused on investigating the mechanisms of obesity and its complications through gut research; the middle stage concentrated on the impact of intestinal inflammation and gut microbiota on obesity onset and progression; and the recent stage emphasized the development of specific microbiota or metabolites and the role of certain immune cell populations in the development of obesity.

**Conclusion:**

Over the past 20 years, research on intestinal immunity and obesity has experienced the initial rapid expansion, stabilization period, and current breakthrough period. The in-depth application of multi-omics analysis and artificial intelligence, as well as the development of gene editing technology, may provide new ideas for targeted modulation of specific intestinal immune cells or microbes for obesity treatment, which may be the main direction of future research in this field.

## 1. Introduction

In recent years, the global prevalence of obesity has increased continuously. The World Obesity Federation predicts that by 2035, 54% of the global population will be overweight or obese.^[1]^ An analysis of Global Burden of Disease data from 2000 to 2019 reveals that among the 5 metabolic disorders, including hypertension, type 2 diabetes, dyslipidemia, obesity, and metabolic-associated fatty liver disease, obesity has the highest standardized mortality rate and disease burden.^[2]^ Moreover, obesity serves as an independent risk factor that increases the incidence and mortality rates of chronic diseases such as type 2 diabetes, coronary heart disease, and stroke.^[3]^ Recent studies have recognized chronic inflammation as the pathological basis for the development of obesity-induced diseases.^[4]^ Therefore, it is crucial to investigate the factors driving chronic inflammation in tissues and organs under obese conditions to identify potential therapeutic targets for obesity.

The intestine is a crucial organ responsible for digestion, nutrient absorption, and immune defense and, playing a vital role in regulating energy metabolism and inflammatory responses.^[5]^ The intestinal mucosa, which contains numerous immune cells and a series of aggregated or isolated lymph glands, interacts with epithelial cells and gut microbiota, forming the largest mucosal immune barrier in the body.^[6]^ It is demonstrated that intestinal immune dyshomeostasis may induce chronic inflammatory response in obese conditions. Clinical studies have revealed that under obese conditions, intestinal adaptive immune cell subsets shift toward a pro-inflammatory phenotype, with elevated levels of inflammatory cytokines.^[7]^ Animal experiments have demonstrated that chronic high-fat diet intake could induce the gut microbiota dysbiosis, disrupt the proportion of innate and adaptive immune cells, and lead to intestinal inflammation, ultimately damaging the gut barrier. As a result, pro-inflammatory cytokines and pathogen-associated molecular patterns, such as lipopolysaccharides (LPS), peptidoglycans, and flagellins, could penetrate the impaired gut barrier into the blood circulation and adipose tissue, thereby inducing or exacerbating systematic metabolic inflammation.^[5,8,9]^

The relationship between intestinal immunity and obesity has recently become a critical research focus in metabolic disease research. Although numerous studies have investigated its pathological mechanisms and therapeutic interventions, systematic sorting and clear presentation of research findings in this field are still lacking. Bibliometric analysis is a quantitative method using mathematical and statistical tools to assess literature performance and visualize research trends.^[10]^ Therefore, this method could effectively illustrate the development trends of a certain research field and the evolution of research frontiers.

This study employed bibliometric analysis and knowledge graph visualization to systematically review the research literature on the relationship between intestinal immunity and obesity from 2004 to 2024. This study aims to address the following research questions:

1. Which authors, institutions, and countries have made significant contributions to the research on the relationship between intestinal immunity and obesity?
2. How have the research focuses and themes evolved over time in the field of the relationship between intestinal immunity and obesity, and what are the characteristics of each stage?

To answering these questions, this study will present a clear and comprehensive overview of the literature on the relationship between intestinal immunity and obesity. Furthermore, the findings will offer valuable insights into the research frontier and present new perspectives for future research in this field.

## 2. Materials and methods

### 2.1. Data collection

We utilized Web of Science for literature search. It is one of the most comprehensive databases of academic journals and research articles and is highly suitable for bibliometric analysis.^[11]^ The literature search, completed on May 8, 2024, employed the following strategies for initial search: TS = (“gut” OR “bowl” OR “intestin*” OR “gastrointestin*”) AND TS = (“immune” OR “immunity”) AND TS = (“obesity” OR “obese” OR “overweight”). Publication dates ranged from January 1, 2004 to May 8, 2024. Only English-language papers categorized as articles or review articles were considered.

Exclusion criteria: (1) duplicate articles; (2) studies irrelevant to the topic of intestinal immunity and obesity; (3) articles categorized as abstracts, editorials, conference proceedings, meeting reports, letters, book chapters, retracted publications, or expressions of concern; (4) articles without an abstract or without full-text access; (5) articles focusing on veterinary medicine and aquaculture.

**Figure 1.**
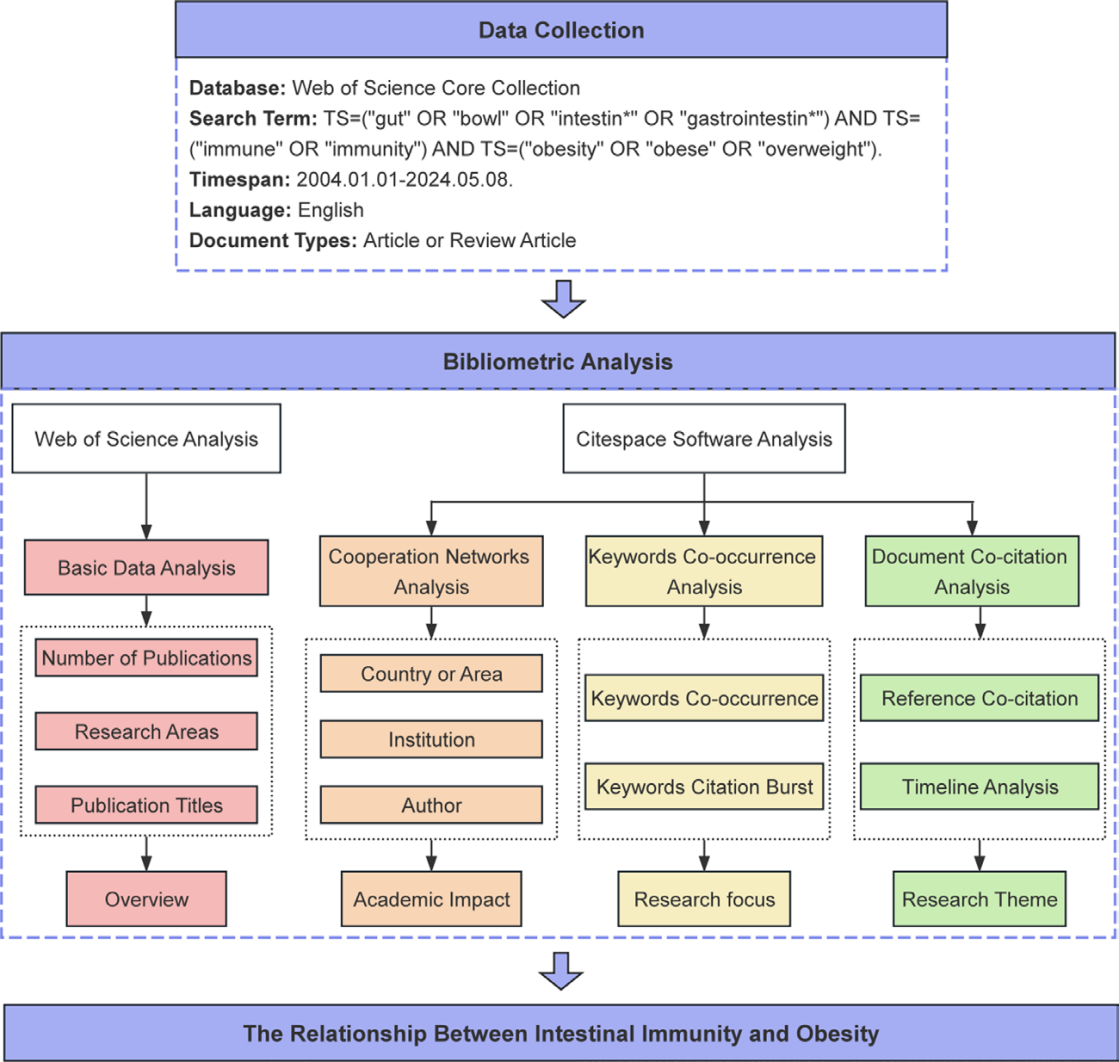
Flowchart depicting the technical process of this study.

All literature retrieval and data acquisitions were finalized on May 8, 2024. After receiving standardized training, 2 researchers, Shanshan Cui and Shuai Shang, independently carried out the data retrieval and cleaning. When disagreements arose regarding article inclusion between the 2 reviewers, a third researcher, Zihui Yan, intervened to arbitrate until a consensus was achieved. Our initial search selected 7225 articles. Through the strict adherence to inclusion and exclusion criteria, a total of 3333 articles were ultimately obtained for further analysis. Key information about these 3333 articles, including titles, authors, keywords, abstracts, countries, institutions, and references, was extracted in TXT format for our bibliometric analysis (Fig. 1).

### 2.2. Data analysis and visualization

GraphPad Prism 8 (GraphPad Software, Boston ) was employed to visualize the 3333 final-selected articles, presenting the trends of publication counts, the global distribution of the top 10 research areas and journals by publication volumes, and the annual publication and citation trends for the top 10 countries in this field. Gephi and Charticulator (Microsoft Research, Redmond) were adopted to generate international collaboration chord graphs in this field.

We conducted detailed data mining and visualization using CiteSpace (version 6.3.R3), an information visualization tool developed by Professor Chen Chaomei, which could transform complex citation networks into explicit knowledge maps.^[12]^ In a map, nodes represent entities (e.g., country, institution, author, keywords, and references), and links between nodes refer to connections and collaborations. The node size indicates the citation or occurrence frequency, and the annual ring color of each node reflects the corresponding year. The betweenness centrality of nodes indicates how frequently a node bridges other nodes through the shortest network pathways. The nodes with high centrality (>0.1) act as important connectors between clusters and are highlighted by the outermost purple ring, whose thickness is proportional to the centrality value. Additionally, modularity (*Q* value) and average silhouette (*S* value) are key criteria for the structure and quality of cluster maps. *Q* value above 0.3 signifies the clear and significant clustering structure, while the *S* value exceeding 0.5 indicates reasonable clustering and *S* value above 0.7 reflects high clustering reliability.

In this study, we used CiteSpace to generate the collaboration network maps for authors, institutions, and countries, thereby identifying leading researchers, core research groups, and influential countries in the field of the relationship between intestinal immunity and obesity. In addition, keyword co-occurrence analysis was performed in CiteSpace to determine the evolution of the research focus in this field. Additionally, reference co-citation analysis and timeline analysis were conducted to reveal the evolution trends of research themes, summarize the characteristics of each developmental stage, and explore emerging research frontiers.

Specific selection criteria were applied to each part of the analysis based on varying analytical perspectives, with details provided in Table 1. For the collaboration network analysis, the *g*-index (*k* = 25) was adopted to evaluate the academic impact of publications in the field of the relationship between intestinal immunity and obesity and thus identify key academic contributors and leading scholars in the research field. In both collaboration network analysis and document co-citation analysis, top N = 30 was implemented to analyze the top 30 nodes with the highest frequency or citation count in each time period. For the keywords co-occurrence analysis, top N = 40 was applied to expand the dataset, selecting the 40 most frequently occurring keywords in each period.

**Table 1:** Parametric settings of CiteSpace software for bibliometric analysis.

Parameters	Specified values
Time slicing	From 2004 Jan to 2024 May, years per slice = 1
Term source	Title, Abstract, Author Keywords (DE), Keywords Plus (ID)
Node types	Author, Institution, Country, Keyword, Reference
Links type	Strength: Cosine, Scope: within slices
Selection criteria	Scale factor *k* of *g*-index = 25 Top N = 30 (collaboration network analysis and document co-citation analysis) Top N = 40 (keywords co-occurrence analysis)

All data used in this research were obtained from publicly accessible database Web of Science. As this research only involves secondary analysis of published research data, additional ethical approval is not required.

## 3. Results

### 3.1. Bibliometric overview

**Figure 2.**
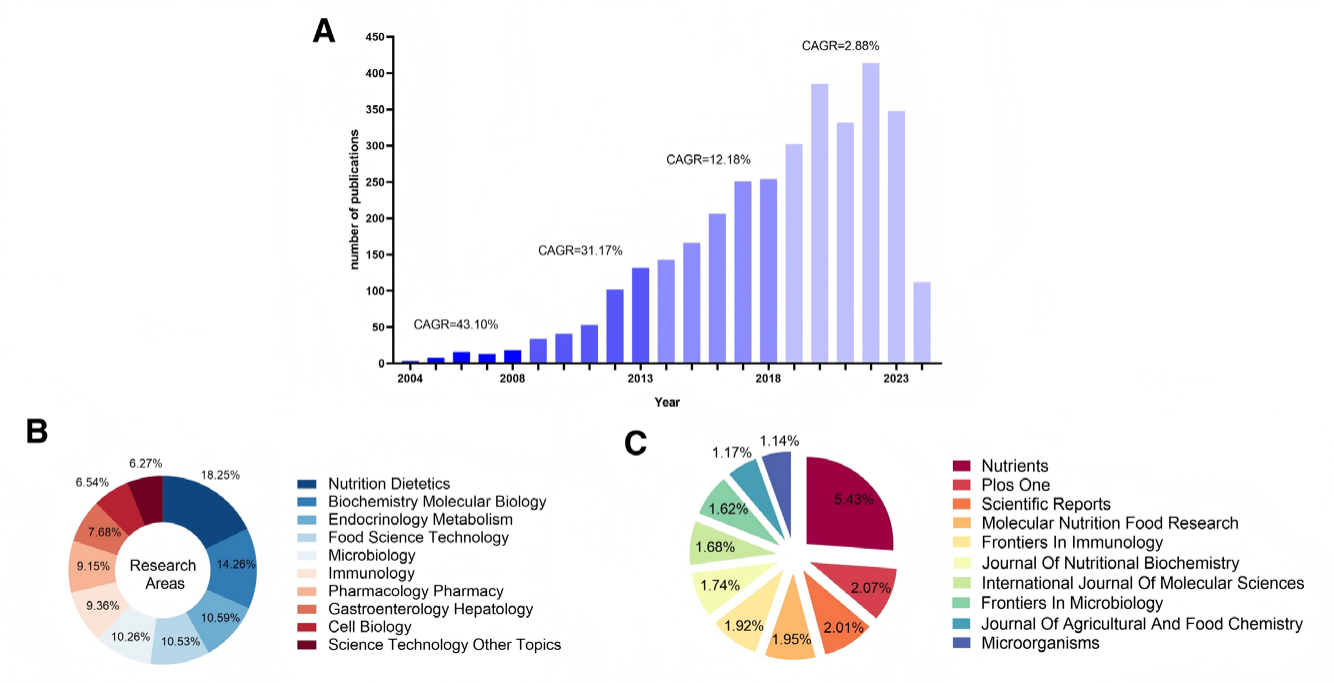
(A) Trends in the number of publications for research in the intestinal immunity and obesity from 2004 to 2024. The compound annual growth rate (CAGR) demonstrates the geometric mean of the annual publication growth rates over the indicated periods. (B) The proportions of the top 10 research areas contributing to this body of literature. (C) Distribution of the top 10 publication titles from 2004 to 2024.

Figure 2A shows a general upward trend from 2004 to 2024 in the publication counts concerning the relationship between intestinal immunity and obesity. During 2004 to 2008, the compound annual growth rate (CAGR) peaked at 43.10%. Following this peak, CAGR decreased to 31.7% between 2009 and 2013 but remained robust. Therefore, from 2004 to 2013, this research field was in the early development stage, characterized by burgeoning academic passion and a surge in annual publication counts. Publication growth moderated during 2014 to 2018, signifying a stable academic growth phase. Between 2019 and 2023, CAGR further declined to 2.88% with significant fluctuations in annual publication counts, potentially due to the COVID-19 pandemic, reduced research funding, or shifts in research focus.

Research on the relationship between intestinal immunity and obesity spanned multiple disciplines (Fig. 2B), with *Nutrition and Dietetics* accounting for the largest proportion (18.25%), followed by *Biochemistry and Molecular Biology* (14.26%). A total of 3333 articles were published in 845 academic journals. Figure 2C reveals that *Nutrients* ranked first among the top 10 journals by publication count (5.43% of final-selected articles), while the other 9 journals each accounted for approximately 1%–2%, reflecting the broad coverage and balanced distribution of journals publishing research in this field. Notably, the top 10 journals corresponded closely to the top 10 disciplines, signifying that research on the relationship between intestinal immunity and obesity concentrated on the areas of nutrition, food science, immunology, and microbiology.

### 3.2. Collaboration network analysis

A total of 3333 publications were analyzed in this study, involving 16,144 authors and 3372 research institutions from 97 countries or regions.

#### 3.2.1. Counties and regions

**Figure 3.**
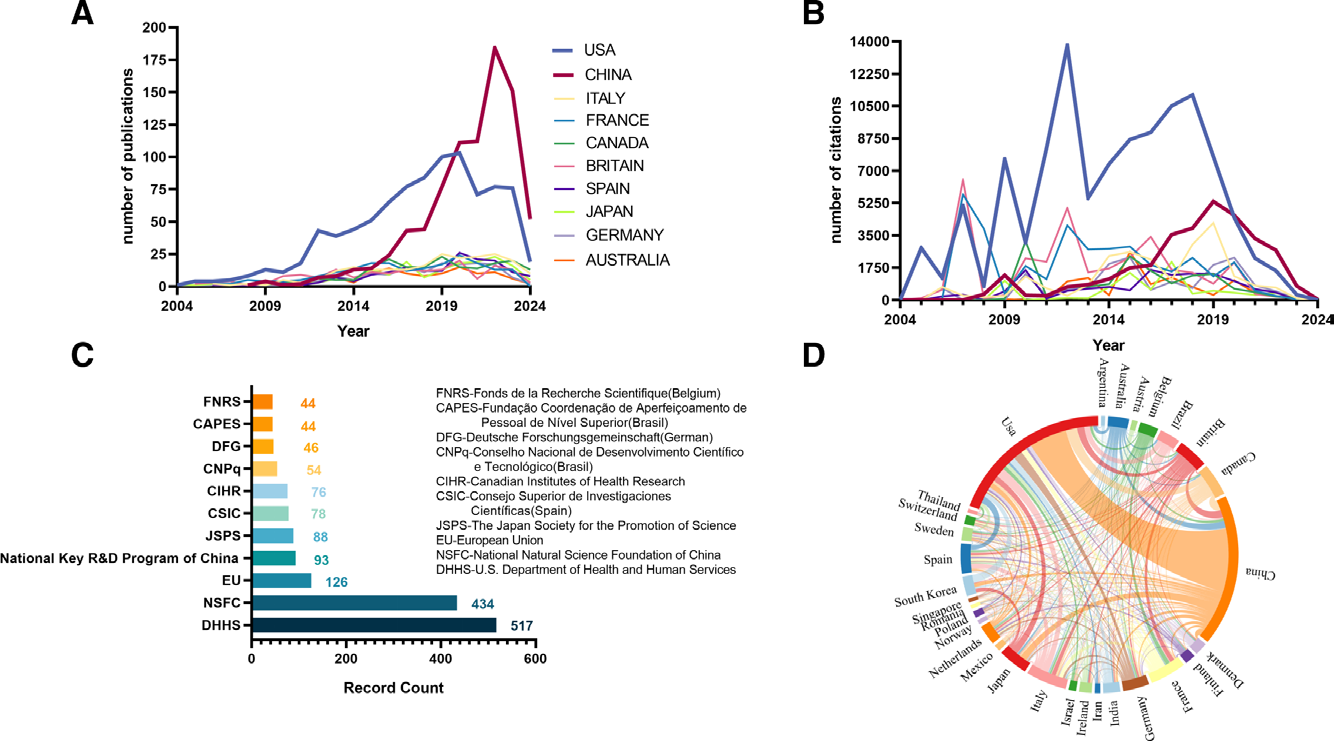
(A) The number of interannual publications in the top 10 countries. (B) The number of interannual citations in the top 10 countries. (C) Top 10 organizations in terms of number of grants. (D) Global collaboration in the field of intestinal immunity-obesity research.

Table 2 displays the top 10 countries by publication count in the research field on the relationship between intestinal immunity and obesity. The United States ranked first with 913 publications (27.39%), followed by China with 848 publications (25.44%). The United States also had the highest total citation count (111,281). Remarkably, China’s annual publication counts and annual total citations have increased significantly in the past decade and surpassed those of the United States in 2020, making China a key contributor in this field (Fig. 3A and B). The U.S. Department of Health and Human Services was the most prominent funding agency in the field, supporting research 517 times. Among the top 10 funding institutions, 2 were based in China: the National Natural Science Foundation of China (434 times) and the National Key R&D Program of China (93 times) (Fig. 3C). Figure 3D presents an intricate global collaboration network in the research on the relationship between intestinal immunity and obesity, with the United States (centrality: 0.38), Britain (0.29), and China (0.23) serving as essential nodes to interconnect with other clusters and promote interregional academic collaboration.

**Table 2:** Top 10 countries with publications in the field of intestinal immunity-obesity research.

Rank	Country	Counts (%)	Citations	Avg. citations	Centrality
1	USA	913 (27.39)	111,281	121.88	0.38
2	China	848 (25.44)	32,686	38.54	0.23
3	Italy	228 (6.84)	22,695	99.53	0.23
4	France	202 (6.06)	35,292	174.71	0.11
5	Canada	182 (5.46)	14,715	80.85	0.02
6	Britain	171 (5.13)	32,453	189.78	0.29
7	Spain	167 (5.01)	12,952	77.55	0.11
8	Japan	166 (4.98)	8261	49.76	0.01
9	Germany	147 (4.41)	15,651	106.46	0.04
10	Australia	117 (3.51)	9928	84.85	0.03

#### 3.2.2. Institutions

**Figure 4.**
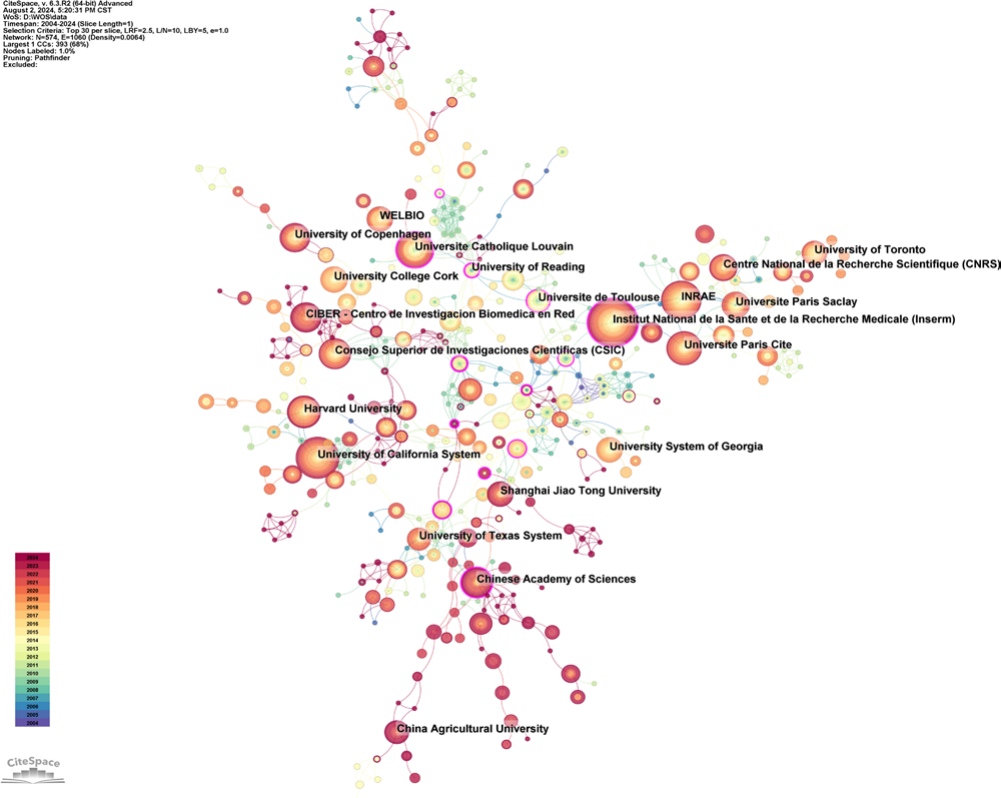
The cooperation networks of institution. Collaboration network analysis utilized a *g*-index (*k* = 25), top N (N = 30), pathfinder, pruning the merged network. In this visualization, each node symbolizes an institution, with lines denoting academic collaborations. Node size reflects the institution’s publication volume. Nodes with a purple outer ring highlight those with high centrality (above 0.1), indicating their prominence in the academic network. Purple rings also reflect their activity level and extensive cooperative relationships.

Figure 4 shows the global collaboration network of institutions, and Table 3 exhibits the top 10 institutions by publication count and centrality. In Table 3, Institut national de la santé et de la recherche médicale (INSERM) held the top position with 127 publications, accounting for 3.81% of the total analyzed literature. As illustrated in Figure 4 and Table 3, INSERM formed a cooperative cluster with INRAE and Université Paris Cité, the former ranking third with 80 publications (2.40%), and the latter ranking sixth with 63 publications (1.89%). All 3 intuitions are from France. Additionally, the University of California System ranked second (104 publications, 3.12%), and Harvard University in fourth place (71 publications, 2.13%) were American institutions that tended to form cooperative clusters. In Span, Consejo Superior de Investigaciones Científicas (60 publications, 1.80%) and Centro de Investigacion Biomedica en Red ranked seventh and ninth respectively, and showed convergent collaboration networks. Moreover, institutions with high centrality, such as the University of Reading (centrality: 0.29) in the United Kingdom, Cornell University (0.2) in America, INSERM (0.17) in France, and the Chinese Academy of Sciences (0.16), acted as core nodes within their respective countries, connecting different clusters, and fostering cross-regional collaborations.

**Table 3:** The top 10 institutions in terms of counts and centrality ranking.

Rank	Institution	Counts (%)	Rank	Institution	Centrality
1	INSERM	127 (3.81)	1	University of Reading	0.29
2	University of California System	104 (3.12)	2	Cornell University	0.20
3	INRAE	80 (2.40)	3	INSERM	0.17
4	Harvard University	71 (2.13)	4	Chinese Academy of Sciences	0.16
5	Université catholique de Louvain	64 (1.92)	5	Université de Toulouse	0.14
6	Université Paris Cité	63 (1.89)	6	Baylor College of Medicine	0.14
7	Consejo Superior de Investigaciones Científicas	60 (1.80)	7	University of Illinois Urbana-Champaign	0.14
8	University of Copenhagen	54 (1.62)	8	Université catholique de Louvain	0.13
9	CIBER – Centro de Investigacion Biomedica en Red	52 (1.56)	9	University of North Carolina	0.13
10	Chinese Academy of Sciences	48 (1.44)	10	University of Pennsylvania	0.13

#### 3.2.3. Authors

**Figure 5.**
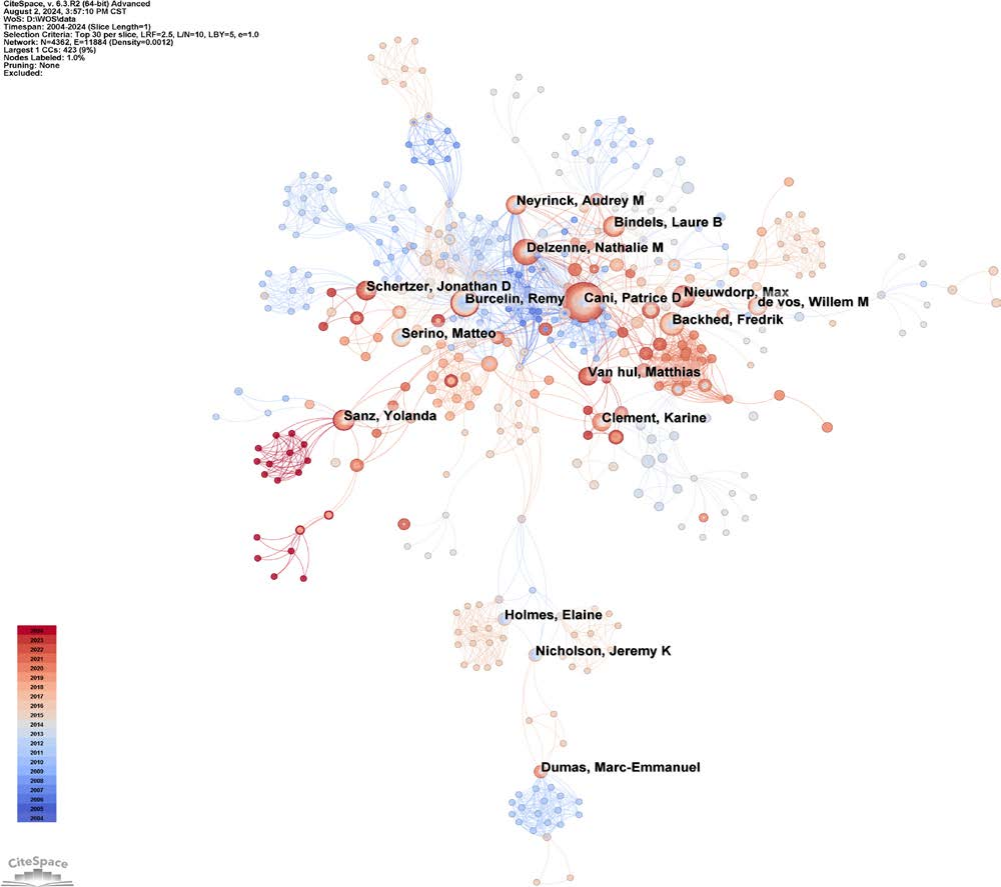
The cooperation networks of authors. Collaboration network analysis utilized a *g*-index (*k* = 25), top N (N = 30), no pruning.

We employed CiteSpace to develop an author collaboration network for the research field on the relationship between intestinal immunity and obesity, selecting 4382 authors and thereby generating 11,884 collaborative relationships (Fig. 5). In Table 4, of the top 10 authors by publications, Patrice D. Cani ranked first with 55 articles and an *H*-index of 109, indicating his most significant influence on this research field. Moreover, the second-ranked author Nathalie M. Delzenne (33 publications, *H*-index: 2), the seventh-ranked author Audrey M. Neyrinck (15 publications, *H*-index: 3), the tenth-ranked author Laure B. Bindels (14 publications, *H*-index: 1) and Patrice D. Cani all affiliated with the Université catholique de Louvain in Belgium.

**Table 4:** Top 10 authors with publications in the field of intestinal immunity-obesity research.

Rank	Author	Counts (%)	*H*-index	Institution	Country
1	Cani, Patrice D	55 (1.65)	109	Université catholique de Louvain	Belgium
2	Delzenne, Nathalie M	33 (0.99)	2	Université catholique de Louvain	Belgium
3	Burcelin, Remy	21 (0.63)	18	INSERM	France
4	Backhed, Fredrik	18 (0.54)	104	University of Gothenburg	Sweden
5	Nieuwdorp, Max	17 (0.51)	70	University of Amsterdam	Netherlands
6	Sanz, Yolanda	16 (0.48)	47	Consejo Superior de Investigaciones Científicas	Spain
7	Neyrinck, Audrey M	15 (0.45)	3	Université catholique de Louvain	Belgium
8	Chassaing, Benoit	14 (0.42)	50	Georgia State University	USA
9	Schertzer, Jonathan D	14 (0.42)	46	McMaster University	Canada
10	Bindels, Laure B	14 (0.42)	1	Université catholique de Louvain	Belgium

In the author collaboration network (Fig. 5), temporal visualization was achieved through color gradients from blue to red within the nodes, revealing consistently active contributors and emerging authors in this field. For instance, Cani and Delzenne have been active since 2007, whereas Burcelin and Backhed have decreased their outputs over the past 5 years. In the last 5 years, Nieuwdorp, Schertzer, and Chen have emerged as more active contributors. Notably, only Cani showed a centrality above 0, suggesting a relative lack of authors who serve as highly central connectors in this field.

### 3.3. Keywords co-occurrence analysis

**Figure 6.**
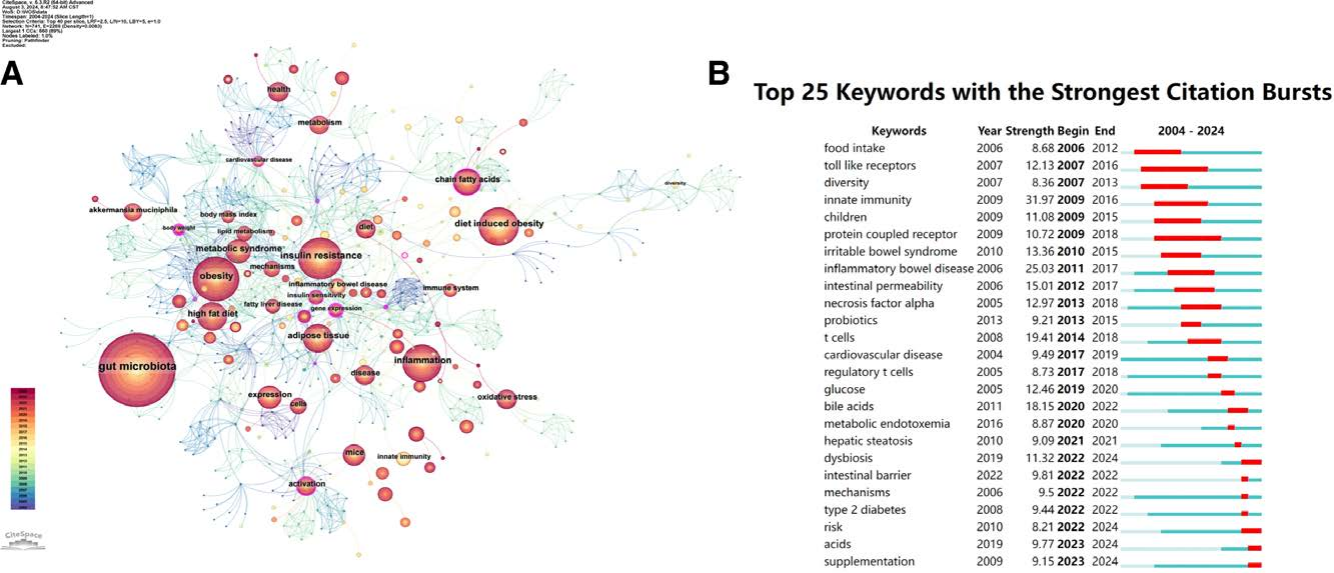
(A) Keywords co-occurrence network. Collaboration network analysis utilized a *g*-index (*k* = 25), top N (N = 40), pathfinder, pruning the merged network. (B) Top 25 keywords with the strongest citation bursts. The frequency of keyword occurrences varies over time, with the light blue line segment indicating that an article has not yet been published and the dark blue line segment depicting when an article was published. The beginning of the red line segment marks the start of the emergence cycle, while the end of the red line segment marks the end of the emergence cycle.

Figure 6A presents a keyword co-occurrence network with 741 keywords and 2269 links. The top 5 keywords by frequency were “gut microbiota” (frequency: 2086), “obesity” (815), “insulin resistance” (749), “inflammation” (612), and “diet induced obesity” (608). Keywords with high centrality (>0.1) serve as bridges linking various keyword clusters. In Table 5, high-centrality keywords “blood pressure” (centrality: 0.21), “cardiovascular disease” (0.13), and “central nervous system” (0.13) acted as important links between obesity research and studies on vascular and neurological complications of obesity.

**Table 5:** Top 10 keywords with the highest frequency and highest centrality.

Rank	Keywords	Frequency	Year	Rank	Keywords	Centrality	Year
1	gut microbiota	2086	2005	1	body weight	0.28	2005
2	obesity	815	2006	2	gene expression	0.23	2005
3	insulin resistance	749	2005	3	blood pressure	0.21	2005
4	inflammation	612	2008	4	activation	0.19	2005
5	diet induced obesity	608	2008	5	insulin sensitivity	0.13	2005
6	high fat diet	396	2005	6	cardiovascular disease	0.13	2004
7	adipose tissue	392	2005	7	central nervous system	0.13	2005
8	metabolic syndrome	351	2006	8	blood mononuclear cells	0.13	2005
9	chain fatty acids	329	2008	9	chain fatty acids	0.12	2008
10	mice	290	2005	10	weight loss	0.11	2005

Furthermore, we performed a citation-burst analysis of keywords (Fig. 6B) to examine developmental trends and attention levels of research in the field of the relationship between intestinal immunity and obesity from 2004 to 2024. Figure 6B shows the top 25 keywords with the strongest citation bursts. The keyword “innate immunity” ranked first with the highest burst intensity (31.97) and the longest burst duration were maintained by “toll like receptors” (from 2007 to 2016) and “protein coupled receptor” (from 2009 to 2018).

In the initial 5 years (2004–2008), keywords like “food intake,” “toll like receptors,” and “diversity” had high citation intensity. In the subsequent period of 2009 to 2013, the number of keywords with strong citation intensity increased to 9, predominantly related to intestinal inflammation and permeability, such as “innate immunity” and “irritable bowel syndrome.” Then in the 2014 to 2018 period, only 3 keywords like “t cells” and “regulatory t cells” emerged with high citation intensity, reflecting the shift of research focuses to T-cell-mediated immunity. The most recent period (2019–2023) witnessed the highest number of keywords (12) with high citation intensity. The emergence of keywords like “bile acids,” “acids,” “dysbiosis,” and “intestinal barrier” illustrated the increasing research interest in gut microbiota and the intestinal barrier. Meanwhile, the keywords “glucose,” “metabolic endotoxemia,” “type 2 diabetes” reflected that obesity-related diseases was another research focus in this period. Remarkably, there was a trend toward a shorter average duration of research focus from 2004 to 2024. The average duration decreased from 8 years in the 2004 to 2008 period, 6 years in the next period (2009–2013), 3 years in the 2014 to 2018 period, and further to approximately 1 year during 2019 to 2023. The 2019 to 2023 period featured proliferating keywords with high citation intensity, indicating a shift to periods with shorter yet more intensive research attention.

### 3.4. Co-citation reference networks

**Figure 7.**
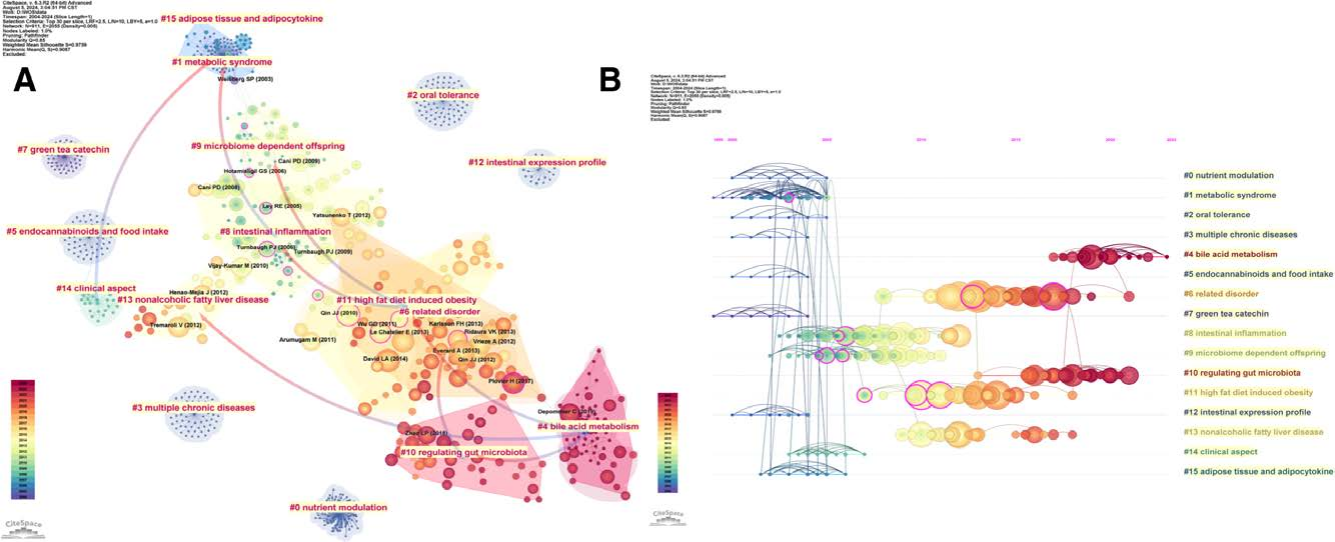
(A) Co-citation document network. Collaboration network analysis utilized a *g*-index (*k* = 25), top N (N = 30), pathfinder, pruning the merged network. Arrows in this figure shows the mutual dependencies among different clusters, where the arrow points to the cluster serving as the knowledge foundation for the other. (B) Clustering time plot of co-citation reference. Clusters were formed using a clustering algorithm based on noun phrases from reference titles, grouping citations with similar subject matter. These clusters are displayed along a timeline from left to right, with earlier (darker) and more recent (lighter) publications. Labels for each cluster are positioned on the timeline’s right side, with the color of these labels indicating the average publication year of the citations within that cluster. Nodes connected by lines indicate co-citation relationships, where linked references are cited together in the same or multiple works. Purple nodes highlight clusters with higher centrality in the network.

Figure 7A presents a reference co-citation network from the final-selected literature. With the value of *Q* = 0.85 > 0.3, and the value of *S* = 0.9759 > 0.7, the network achieves very clear and highly reliable clustering. Based on the LLR algorithm and cluster labels extracted from reference titles, 16 co-citation clusters were extracted and numbered from 0 to 15 in descending order of size. The arrows in Figure 7A indicate the citation relationships among various co-citation clusters, revealing the mutual dependencies and evolutionary trajectories of the research themes. For instance, the literature in cluster #1 “metabolic syndrome” provided the knowledge base for cluster #8 “intestinal inflammation,” cluster #14 “clinical aspect,” and cluster #15 “adipose tissue and adipocytokine.” Likewise, cluster #8 “intestinal inflammation” and cluster #9 “microbiome dependent offspring” provided a research foundation for cluster #11 “high fat diet induced obesity.” Additionally, cluster #10 “regulating gut microbiota” cited research from cluster #6 “related disorder,” and cluster #4 “bile acid metabolism” built on clusters #10 “regulating gut microbiota,” cluster #11 “high fat diet induced obesity,” and cluster #13 “nonalcoholic fatty liver disease.”

The top 10 co-cited references were listed in Table 6, where *Cross-talk between Akkermansia muciniphila and intestinal epithelium controls diet-induced obesity*^[13]^ ranked first as the most frequently co-cited reference. This literature was published in 2013 with Patrice D. Cani as the corresponding author.

**Table 6:** Top 10 of research articles with the highest number of co-citation.

Rank	References	Counts	Centrality	Year	Cluster
1	Everard A, Belzer C, Geurts L, et al. Cross-talk between *Akkermansia muciniphila* and intestinal epithelium controls diet-induced obesity. *PNAS*. 2013;110(22):9066–9071.	165	0.01	2013	#6
2	David LA, Maurice CF, Carmody RN, et al. Diet rapidly and reproducibly alters the human gut microbiome. *Nature*. 2014;505(7484):559–563.	146	0.05	2014	#11
3	Qin J, Li Y, Cai Z, et al. A metagenome-wide association study of gut microbiota in type 2 diabetes. *Nature*. 2012;490(7418):55–60.	140	0.05	2012	#6
4	Ridaura VK, Faith JJ, Rey FE, et al. Gut microbiota from twins discordant for obesity modulate metabolism in mice. *Science*. 2013;341(6150):1241214.	130	0.01	2013	#6
5	Qin J, Li R, Raes J, et al. A human gut microbial gene catalogue established by metagenomic sequencing. *Nature*. 2010;464(7285):59–65.	123	0.15	2010	#11
6	Le Chatelier E, Nielsen T, Qin J, et al. Richness of human gut microbiome correlates with metabolic markers. *Nature*. 2013;500(7464):541–546.	121	0.10	2013	#6
7	Turnbaugh PJ, Hamady M, Yatsunenko T, et al. A core gut microbiome in obese and lean twins. *Nature*. 2009;457(7228):480–484.	117	0.02	2009	#8
8	Tremaroli V, Bäckhed F. Functional interactions between the gut microbiota and host metabolism. *Nature*. 2012;489(7415):242–249.	115	0.01	2012	#13
9	Plovier H, Everard A, Druart C, et al. A purified membrane protein from *Akkermansia muciniphila* or the pasteurized bacterium improves metabolism in obese and diabetic mice. *Nat Med*. 2017;23(1):107–113.	114	0.10	2017	#6
10	Vijay-Kumar M, Aitken JD, Carvalho FA, et al. Metabolic syndrome and altered gut microbiota in mice lacking Toll-like receptor 5. *Science*. 2010;328(5975):228–231.	107	0.05	2010	#13

By organizing 16 clusters chronologically in Figure 7A, the timeline graph of co-citation reference clusters (Fig. 7B) was constructed with cluster numbers on the *Y*-axis and citation publication years on the *X*-axis. Based on the average publication year of each cluster, historical research in this field could be classified into 3 stages: early (2000–2004), middle (2005–2013), and recent (2014–present). The early stage included clusters #7, #12, #0, #2, #3, #5, #1, and #15; the middle stage comprised clusters #14, #9, #8, #11, and #13; and the recent stage (2014–2024) comprised clusters #6, #10, and #4. By analyzing co-citation reference clusters and their relationships at each stage, this study will reveal the evolution of research themes in the field of the relationship between intestinal immunity and obesity, providing insights into future research directions.

## 4. Discussion

Obesity mainly results from an imbalance in energy metabolism in which dietary energy intake exceeds energy expenditure through physical activity. A pathological feature of obesity is low-grade, chronic systemic inflammation. The intestine serves not only as a crucial digestive organ, but also as the largest immune organ. Recent findings indicate that the intestinal immune system is essential for maintaining gut barrier integrity and for regulating immune tolerance and inflammatory responses.^[14]^ It also engages in complex crosstalk with enteroendocrine cells to modulate nutrient absorption and metabolism.^[15]^ Furthermore, intestinal immunity is closely connected with the gut microbiota, the latter regulates the development, differentiation, and physiological functions of intestinal immune cells.^[16]^ Given its unique anatomical and functional role in regulating energy metabolism, the intestinal immune system has been a key target in obesity research, attracting substantial research interest in elucidating its relationship with obesity. To comprehensively view the historical development of the relationship between intestinal immunity and obesity, we conducted a bibliometric analysis and knowledge graph visualization of the literature on this field from 2004 to 2024, investigating influential researchers, institutions, and countries, identifying major research focuses, and tracing theme evolution in this field.

### 4.1. Influential countries, institutions, and researchers

In the field of the relationship between intestinal immunity and obesity, the United States maintained its top position in both total publication output and total citation counts for a long time. American research institutions and scholars also played a crucial role in international cooperation and academic exchange in this field, which may be attributed to the country’s early research engagement and substantial government funding. However, in 2020, China surpassed the United States in terms of annual publication count and annual total citations. Represented by the Chinese Academy of Sciences, Chinese research institutions and scholars, although entered this field later than the United States, were increasingly active in national and international collaborations and had a growing academic influence on the field. Such a surge in research output and citations is likely to be caused by substantial Chinese government funding, particularly through the National Natural Science Foundation of China and the National Key R&D Program. Nevertheless, the average citation count from China remained relatively low, indicating its limited research influence. Although possibly due to the later start of the field in China, this also implies the need for more high-quality publications in high-impact journals.

Globally, research institutions in the field of the relationship between intestinal immunity and obesity tended to form regional research clusters, with closer collaboration among institutions from the same country or geographically adjacent regions. For instance, INSERM, a French institution that ranked first in publication counts, formed the close cooperative cluster with INRAE and Université Paris Cité, both also based in France, making significant contributions to research advancements in this field. Additionally, INSERM had high centrality, reflecting its pivotal role as a core research institution in facilitating cross-cluster academic exchanges and international collaboration.

As for influential authors, Patrice D. Cani played a leading role in the field of the relationship between intestinal immunity and obesity. He has been an active researcher since 2007, and has continued to contribute to this field. The *Cross-talk between Akkermansia muciniphila and intestinal epithelium controls diet-induced obesity*,^[13]^ where he served as the corresponding author, was the most frequently co-cited reference in this field. This study demonstrated that *Akkermansia muciniphila* level is greatly reduced in obese and type 2 diabetic mice, and that its supplementation could mitigate high-fat diet-induced metabolic disorders. As a highly studied bacterial genus in intestine in recent years, *Akkermansia muciniphila* is an anaerobic gut bacterium primarily colonizing in the intestinal mucus layer, with a crucial role in regulating energy metabolism and mucosal immunity.^[17]^

Additionally, Cani, coauthored multiple studies with Delzenne and colleagues at his institution, making significant contributions to this field. This team was the first to identify that gut microbiota-induced intestinal barrier dysfunction is a potential mechanism underlying metabolic inflammation and high-fat diet-induced obesity.^[18–20]^ Their subsequent research further investigated the relationships between gut microbiota, intestinal immunity, and metabolic diseases. Their research found that under pathological conditions, gut microbiota and their metabolites could influence the proportion of certain gut immune cells to induce intestinal inflammation and impair the immune barrier, which enables pro-inflammatory cytokines and pathogen-associated molecular patterns to enter the circulation, leading to low-grade systematic inflammation and ultimately causing metabolic diseases.^[21,22]^ Furthermore, Remy Burcelin conducted an adoptive transfer experiment with Cani’s team, demonstrating that gut microbiota could modulate metabolic phenotypes by regulating the expression and function of retinoic acid-related orphan receptor γt + cluster of differentiation 4+ T cells (termed as T helper cell 17) in the intestine.^[23]^ Despite the author collaboration network in the field comprising 4362 authors and 11,884 collaborative relationships, highly central authors are still lacking in this field, probably because most researchers tend to team within single research groups from the same research institution or country rather than collaborating across teams and regions.

### 4.2. The evolution of research focuses

Keyword co-occurrence analysis revealed that research on the relationship between intestinal immunity and obesity from 2004 to 2024 featured the shorter research focus duration, and the proliferation and rapid shift of research focuses, signifying the growing academic attention to this field. Regarding the research keywords, “innate immunity,” “toll-like receptors,” “T cells,” “regulatory T cells,” “tumor necrosis factor-alpha,” “intestinal barrier,” “intestinal permeability,” “metabolic endotoxemia,” “intestinal dysbiosis,” “probiotics,” and “bile acids” could be divided into 3 major categories: immunity, intestinal barriers, and gut microbiota, which are directly related to the intestinal immune system and involved in the pathogenesis of obesity. Moreover, keywords such as “irritable bowel syndrome,” “inflammatory bowel disease,” “cardiovascular disease,” “hepatic steatosis,” and “type 2 diabetes” are obesity-related diseases, whose pathophysiology is linked to intestinal immunity. Therefore, this study will discuss the evolution of the research focuses from 2004 to 2024 from 2 perspectives: the pathogenesis of obesity and obesity-related diseases.

#### 4.2.1. The pathogenesis of obesity

Studies have shown that long-term high-fat diets could alter gut microbiota composition and disrupt intestinal immune balance, causing intestinal barrier dysfunction and increased permeability. This enables pathogenic gut microbial metabolites, such as LPS and inflammatory cytokines, to enter the circulation, triggering low-grade chronic systemic inflammation that consequently leads to obesity.^[18,24,25]^ Therefore, the intestinal immune system and gut microbiota are crucial in the pathological course of high-fat diet induced obesity. Over the past 2 decades, intestinal immunity and gut microbiota has received relatively high attention with continuous achievements. This section will elucidate the evolution of research focuses on the pathogenesis of obesity from 2 perspectives: intestinal immunity and gut microbiota.

##### 4.2.1.1. From innate immunity to adaptive immunity

In the early stage of the research on the relationship between intestinal immunity and obesity, “innate immunity” was the keyword with the highest citation intensity. The innate immunity of the gut, comprising the intestinal barrier, innate immune cells, and cytokines, forms the first line of defense against pathogenic invasion.^[26]^ Studies find that long-term high-fat diet intake could cause gut microbiota dysbiosis, disrupt tight junctions between epithelial cells, and increase epithelial apoptosis, ultimately leading to intestinal barrier dysfunction.^[24,25]^ Meanwhile, intestinal environment changes and the exposure to pro-inflammatory agents such as LPS, could induce macrophage polarization and increase the proportion of M1 macrophages. These M1 macrophages subsequently release pro-inflammatory cytokines, including interleukin‑6 (IL‑6), IL‑1β, and tumor necrosis factor‑α, which exaggerate local and systemic inflammatory responses and cause low-grade chronic systemic inflammation, ultimately leading to obesity.^[27–29]^ Furthermore, the intestinal innate lymphoid cells (ILCs) also play an important role in the pathogenesis of diet-induced obesity. ILCs are classified into 3 subtypes (ILC1, ILC2, and ILC3) based on the expression of transcription factors expression and cytokines secretion. ILC3 predominantly produces IL‑22, a cytokine that promotes the proliferation of intestinal epithelial cells, maintains tight junctions, and reduces intestinal permeability.^[30]^ Studies have demonstrated that high‑fat diets reduce the proportion of ILC3 cells and IL‑22 secretion, thereby disrupting intestinal immune homeostasis and impairing the repair capacity of the intestinal barriers after injury.^[31]^

In the middle stage, the keywords “T cells” and “regulatory T cells” received the highest citation intensities. “T cells” constitute essential components of the adaptive immunity in the gut. Under continuous antigen stimulation, innate immune cells initiate adaptive immune responses by presenting antigens and secreting cytokines, which, in turn, activates naive T lymphocytes and differentiates them into diverse effector T cell subsets.^[32]^ Studies indicate that during high-fat diet-induced obesity, many adaptive immune cell subsets in the gut shift toward a pro-inflammatory phenotype. This shift could promote the production of inflammatory cytokines, such as tumor necrosis factor‑α and interferon‑γ, and reduce the secretion of cytokines that are beneficial for metabolism and maintaining intestinal barrier integrity, for instance, IL‑22, IL‑10, and immunoglobulins, ultimately leading to disruption of the intestinal barrier and increased permeability.^[5,33]^ In addition, “regulatory t cells” (Tregs), another keyword at this stage, mainly mediate immune tolerance to innocuous antigens. Through the secretion of anti-inflammatory cytokines, Tregs could suppress epithelial apoptosis and promote epithelial cell proliferation, thus helping sustain epithelial homeostasis.^[34]^ Recent studies have elucidated the mechanisms by which intestinal Tregs are involved in high-fat diet‑induced obesity, such as Th17/Treg imbalance.^[35]^

##### 4.2.1.2. Deepening insights into gut microbiota research

There exists an intricate relationship between the gut microbiota and intestinal immunity, in which gut microbiota colonization requires the establishment of immune tolerance, while the gut microbiota and its metabolites regulate the development, differentiation, and activation of intestinal immune cells.^[36,37]^ Such interplay is crucial for maintaining gut immune homeostasis and restoring the metabolic balance. Microbiota is a major research focus in the field of the relationship between intestinal immunity and obesity.

Early gut microbiota research in this field mainly concentrated on the keywords intestinal “dysbiosis” and “probiotics.” High-fat diet-induced intestinal “dysbiosis,” characterized by the decline in beneficial bacteria and the increase in pathogenic bacteria proportion, could impair intestinal immune function, compromise the intestinal barrier, and subsequently make pro-inflammatory cytokines enter into the circulation, ultimately resulting in chronic systemic inflammation and obesity.^[38]^ Correspondingly, the supplementation of exogenous “probiotics” could promote beneficial bacteria colonization, suppress opportunistic pathogens, and modulate hosts’ intestinal mucosal and systemic immune functions, which contributes to reducing intestinal permeability and overcoming obesity.^[39,40]^

With ongoing investigations into the gut microbiota, research focus on the field of the relationship between intestinal immunity and obesity shifted toward gut microbiota metabolites. Since 2020, “bile acids” has been the most frequently cited keyword in this field. Bile acids are hormones synthesized by the liver, metabolized and absorbed in the intestine, subsequently returning to the liver via the portal circulation. During this process, bile acids circulate systemically, bind to and activate specific bile acid receptors to regulate energy metabolism and maintain immune homeostasis.^[41]^ In the intestine, bile acids are converted by intestinal microbes into secondary bile acids, where lithocholic acid (LCA) and its derivatives, including 3‑oxoLCA, isoalloLCA, and isoDCA, are pivotal in regulating cluster of differentiation 4⁺ T cell differentiation, particularly in the differentiation of Th17 and Treg cells.^[42,43]^ Moreover, it is reveled in animal studies that hyocholic acid species could stimulate the synthesis and secretion of glucagon-like peptide-1 in the intestine and reduce blood glucose levels by activating G-protein-coupled bile acid receptor and suppressing farnesoid X receptor.^[44]^

#### 4.2.2. Obesity-related diseases

Obesity is widely recognized as a significant risk factor for multiple diseases. Compared to individuals with a healthy weight, obese individuals have higher risks of developing comorbidities associated with obesity, such as cardiometabolic diseases, digestive diseases, respiratory diseases, neurological diseases, musculoskeletal diseases, infection, and malignancy.^[45]^ Regrading intestinal immune system, it not only plays a crucial role in the pathogenesis of obesity, but is also involved in the pathomechanisms of obesity-related diseases. The following section will discuss the evolution of research focus in the field of the relationship between intestinal immunity and obesity from the perspective of obesity-related diseases.

At the initial stage, intestinal disease, “irritable bowel syndrome” (IBS) and “inflammatory bowel disease” (IBD) were the keywords with the strongest citation bursts. IBD, including Crohn disease and ulcerative colitis, is a chronic, relapsing, nonspecific intestinal inflammatory disorder, whose pathogenesis is closely associated with intestinal immune dysregulation and metabolic disturbances.^[46]^ Epidemiological data show that the prevalence of IBD has increased in parallel with that of obesity in recent decades.^[47]^ A study from the United Kingdom utilized a multivariate Cox regression model, demonstrating that both overall and abdominal obesity are related to an increased risk of IBD, with metabolic disorders, especially inflammation, potentially serving as a mediating factor between them.^[48]^

The other keyword IBS is a chronic condition characterized by functional intestinal disorders, sharing clinical similarities with IBD.^[49]^ Multiple studies have indicated that dietary factors could trigger or exacerbate IBS symptomatology,^[50]^ and low-grade intestinal inflammation may be involved in the pathogenesis of IBS via neuroimmune mechanisms,^[51]^ similar to the pathogenesis of obesity. Additionally, obesity itself is currently recognized as a potential risk factor for IBS, with clinical studies finding a positive correlation between body mass index and the levels of complement C3 and C-reactive protein in IBS patients.^[52,53]^

In the middle stage, the focus on obesity-related diseases in this field expanded to cardiovascular and liver diseases, reflected in the high citation keywords “cardiovascular disease” and “hepatic steatosis.” Obesity could increase the risk of cardiovascular disease development and mortality, possibly related to adipose tissue dysfunction and inflammation associated with obesity. Inflammatory cytokines could promote macrophage infiltration, vascular inflammation, and pro‑atherogenic gene expression, leading to endothelial injury, hemodynamic disturbances, and myocardial remodeling, which ultimately affects cardiac structure and function.^[54]^ Furthermore, recent studies have suggested that the pathogenesis of cardiovascular disease might be associated with high-fat diet-induced intestinal hyperpermeability and the elevated levels of inflammatory substances in the circulation.^[55]^

In addition, there is a complex interplay between the liver and gut, known as the gut‑liver axis, where dietary nutrients, antigens, gut microbiota, and their metabolites could regulate metabolism and immune responses in the intestine and liver.^[56]^ By impairing intestinal barrier integrity and disrupting immune homeostasis, chronic high-fat intake might increase endotoxins and other pathogenic microbial metabolites in the circulation, causing gut-liver axis dysfunction, and ultimately leading to hepatic disorders, alongside with metabolic inflammation and obesity.^[57,58]^

### 4.3. The evolution of research themes

Co-citation reference network analysis is one of the core functions of the CiteSpace software. By examining the citation network of references and the timeline graph of co-citation reference clusters in this field, this research will elucidate the knowledge base and evolutionary trajectory of research on the relationship between intestinal immunity and obesity, thereby suggesting future research directions.

In the early stage (2000–2004), research themes (#7 green tea catechin, #12 intestinal expression profile, #0 nutrient modulation, #2 oral tolerance, #3 multiple chronic, #5 endocannabinoids and food intake, #1 metabolic syndrome, #15 adipose tissue and adipocytokine) ranged widely, laying the foundation for the field of the relationship between intestinal immunity and obesity. Studies about “nutrient modulation” of the immune response and “oral tolerance” discover the crucial role of diets in shaping the intestinal immune system, in which dietary antigens could influence the proliferation, differentiation, and normal function of immune cells.^[59]^ Studies have shown that obese patients tend to increase their intake of carbohydrates and fats, coupled with deficiencies in dietary fiber and trace elements, resulting in malnutrition that adversely affects the growth, development, and function of immune cells.^[60]^ Meanwhile, increased attention was directed toward the “endocannabinoids” system, revealing its extensive distribution across various tissues, including the brain, liver, pancreas, adipose, and gastrointestinal tracts.^[61]^ The endocannabinoids system plays a critical role in regulating processes such as ingestion, glucose metabolism, lipid metabolism, inflammatory responses, and intestinal barrier maintenance, offering novel insights into the interplay between intestinal immunity and obesity.^[13]^

In terms of “adipose tissue,” it acts as an energy reservoir as well as an endocrine and immunological organ. “Adipocytokine” and pro-inflammatory cytokines secreted by immune cells in adipose tissue are key mediators of obesity-related insulin resistance and systematic inflammation.^[62]^ Such cytokines establish a connection between obesity and the intestinal immunity. As cytokines are mainly secreted by white adipocytes, resistin-like molecules (RELMs) can mediate insulin resistance and obesity by regulating insulin signaling pathways and promoting inflammatory responses.^[63]^ Intestinal cells have also been identified as an important source of RELMs, and RELMs are closely associated with intestinal immunity. A study reveals that upregulated expression of RELM gene in colonic epithelial cells drives elevated RELM levels in the serum of obese mice, and the latter is correlated to the degree of insulin resistance.^[64]^ Meanwhile, research has shown that RELM‑β plays a critical role in maintaining colonic barrier function and innate gastrointestinal immunity.^[65]^ In addition, “metabolic syndrome” was another important research theme at this stage. Obesity is a core symptom of metabolic syndrome, and obese patients often suffer from chronic metabolic disease. Notably, research focus at the early stage included “irritable bowel syndrome” and “inflammatory bowel disease,” both of which are intestinal diseases whose pathogenesis involves metabolic disturbances caused by the imbalance of intestinal immune homeostasis.

During the mid-term stage (2005–2013, research themes: #14 clinical aspect, #9 microbiome dependent offspring, #8 intestinal inflammation, #11 high fat diet induced obesity, and #13 nonalcoholic fatty liver disease), early research themes further progressed while new directions emerged. It has become mainstream to investigate the role of gut microbiota in the pathogenesis of metabolic disorders, particularly obesity and diabetes. Numerous studies reveal that the gut microbiota modulates energy metabolic homeostasis through multiple mechanisms, such as by modulating nutrient digestion and absorption, regulating intestinal endocrine and neural signaling pathways, altering gut permeability, and affecting intestinal immunity.^[66,67]^ Additionally, “high fat diet induced obesity” became a critical research theme at this stage, reflecting the growing attention to the pivotal role of the intestine in regulating energy metabolism. “Intestinal inflammation” is identified as one of the major obesity pathogenesis. Research demonstrates that high-fat diets could cause gut barrier dysfunction and immune dyshomeostasis, leading to local inflammation in the intestine and allowing pro-inflammatory cytokines to enter systemic circulation, thereby inducing low-grade systematic inflammation and ultimately resulting in obesity and insulin resistance.^[18,24,25]^ As an important theme in this stage, “nonalcoholic fatty liver disease” is another key component of metabolic syndrome, which is frequently comorbid with obesity. Given the detailed discussion in Section 4.2.2, this will not be elaborated further here.

In the recent stage (2014–2024), research themes (#6 related disorder, #10 regulating gut microbiota, and #4 bile acid metabolism) illustrated by the timeline graph of co-citation reference clusters were closely related to the gut microbiota. Extensive research has revealed the critical role of gut microbiota and its metabolites in the pathogenesis of metabolic diseases. For instance, as mentioned earlier, primary bile acids could be transformed by gut microbiota into a range of secondary bile acids, which, by binding to receptors like G-protein-coupled bile acid receptor and farnesoid X receptor, could help regulate glucose and lipid metabolism and modulate immune functions.^[41–44]^ “Regulating the gut microbiota” through dietary modulation, fecal microbiota transplantation, or probiotic supplementation has been demonstrated to be effective in improving metabolic disorders.^[38–40]^ Considering the close relationship between intestinal microbiota and the intestinal immune system, these findings greatly enrich research on the relationship between intestinal immunity and obesity.

## 5. Limitations

There are also some limitations in this study: First, this research only analyzes English-language literature in the field of the relationship between intestinal immunity and obesity from the single database Web of Science, without encompassing non-English language publications in this field and relevant research from other databases (e.g., PubMed, Scopus). Second, co-citation analysis relies on accumulated citation data over time, which may cause the exclusion of some recently published high-quality papers from the ranks of high-value research due to insufficient citations. To address this, our future research will comprehensively evaluate the quality of literature published within the past year based on the overall influence of the journals, affiliated institutions, and authors. Third, despite our efforts to develop a more comprehensive search strategy (such as using wildcards to retrieve terms that include singular and plural forms, as well as to capture terms derived from the same core morphemes [e.g., “intestin*” and “gastrointestin*”]) some synonymous terms may still have been overlooked, potentially causing the omission of relevant studies.

## 6. Conclusion

In recent years, the prevalence of obesity has continued to rise worldwide, accompanied by an increase in the morbidity and mortality of noncommunicable diseases comorbid with obesity, such as diabetes, coronary artery disease, stroke, and cancer. Therefore, the significant burden of obesity and its comorbidities on public health systems worldwide necessitates the effective strategies to reduce the prevalence of obesity and prevent comorbidities. The intestinal immune system plays a pivotal role in mediating the progression from high-fat diet-induced systemic chronic inflammation to obesity. Through mutual interactions with intestinal epithelial cells and the gut microbiota, the intestinal immune system could help establish and maintain the intestinal barrier and regulate energy metabolism and chronic inflammatory responses, thereby pioneering new directions in metabolic disease research.

This study comprehensively and systematically examined the core findings and fundamental conclusions of research on the relationship between intestinal immunity and obesity over the past 2 decades. In this study, the research focuses, themes, and their evolution were elucidated and analyzed in detail, aiming to help researchers gain a comprehensive understanding of the historical development and future directions in this field.

Overall, research on the relationship between the intestine and obesity had a dynamic developmental trajectory, characterized by an initial rapid expansion, subsequent stabilization period, and the current breakthrough period. Based on the analysis of keyword citation bursts, the evolution of research focus in this field from 2004 to 2024 could be broadly summarized and classified into 2 aspects: the pathogenesis of obesity and obesity-related diseases. Meanwhile, co-citation analysis of references in this research indicated a thematic evolution trajectory in this field: from a broad focus on the role of the intestine in obesity pathogenesis and obesity-related diseases at the early research stage, through a convergence on the role of intestinal inflammation and gut microbiota in the onset and development of obesity at the middle stage, and to a focus on specific microbial populations, metabolites, and certain immune cell populations at the recent stage.

In the era of interdisciplinary integration and innovative development, combining multi-omics analysis with artificial intelligence, along with the application of gene editing technologies, may offer new research directions for the targeted modulation of specific gut immune cell populations or microbiota. These are expected to offer significant therapeutic potential for the treatment of metabolic inflammatory disorders, including obesity and insulin resistance.

## Author contributions

**Conceptualization:** Zihui Yan, Shuai Shang.

**Data curation:** Zihui Yan, Shanshan Cui.

**Formal analysis:** Zihui Yan.

**Investigation:** Zihui Yan, Shuai Shang.

**Methodology:** Zihui Yan, Shanshan Cui.

**Project administration:** Zihui Yan.

**Software:** Zihui Yan.

**Supervision:** Zihui Yan, Shuai Shang.

**Validation:** Zihui Yan, Shanshan Cui, Shuai Shang.

**Visualization:** Zihui Yan, Shanshan Cui.

**Writing – original draft:** Zihui Yan, Shanshan Cui, Shuai Shang.

**Writing – review & editing:** Zihui Yan.
